# Radar Detection-Inspired Signal Retrieval from the Short-Time Fourier Transform

**DOI:** 10.3390/s22165954

**Published:** 2022-08-09

**Authors:** Karol Abratkiewicz

**Affiliations:** Institute of Electronic Systems, Warsaw University of Technology, 00-665 Warsaw, Poland; karol.abratkiewicz@pw.edu.pl

**Keywords:** signal extraction, radar signal processing, time–frequency analysis

## Abstract

This paper presents a novel adaptive algorithm for multicomponent signal decomposition from the time–frequency (TF) plane using the short-time Fourier transform (STFT). The approach is inspired by a common technique used within radar detection called constant false alarm rate (CFAR). The areas with the strongest magnitude are detected and clustered, allowing for TF mask creation and filtering only those signal modes that contribute the most. As a result, one can extract a particular component void of noise and interference regardless of the signal character. The superiority understood as an improved reconstructed waveform quality of the proposed method is shown using both simulated and real-life radar signals.

## 1. Introduction

Non-stationary and multicomponent signals are present in a variety of applications such as radar [1,2], sonar [3], biomedical engineering [4], and others [5,6,7,8]. In all practical cases, signals are corrupted by noise, interference, disturbances, and multipath propagation (the latter is meaningfully observable for wireless systems, radar, and sonar) [9,10]. The difficulties in analysis resulting from signal quality degradation are challenging since particular component extraction is not trivial. The problem with multicomponent signal decomposition stems from several issues. Firstly, the noise is a common problem preventing unequivocal signal analysis and spoils the properties of many techniques for mode extraction. Secondly, real-life signals may have components of a different nature, e.g., harmonic terms, pulse-shape transients, amplitude and frequency modulation (AM-FM), and many others. Thirdly, common relations between terms can also be obstructed, e.g., components can be entangled or occupy the same bandwidth, time frame, or both simultaneously.

A typical approach to component extracting is the TF processing due to the informative character of the result, the possibility to distinguish particular signal terms, and invertibility [11]. In the literature, several attempts in signal retrieval from different TF distributions can be identified concerning the STFT [12,13,14,15], but also other techniques such as the wavelet transform [16], empirical mode decomposition [17,18], and the Wigner-Ville distribution [19]. In the considered problem, the main goal is to detect the component and create a mask around it. After that, a given component can be retrieved using an inverse transformation. However, one cannot extract and reconstruct the component without any information about the signal character, location on a time–frequency plane, and duration. One of the most popular approaches is signal decomposition using vertical synchrosqueezing (VSS), which is based on relocating transform values along the frequency axes to the local instantaneous frequency ridge [20,21]. As a result, one can obtain a sharp and concentrated distribution, facilitating dominant component extraction. However, the principle of the VSS operation relies on shifting the transform values vertically; hence, impulsive signals and spikes are poorly concentrated or even smeared over the plane. The problem was addressed in [22], where the classical, non-concentrated spectrogram was used. In this approach, the idea was to connect spectrogram zeros using Delaunay triangulation. Then, only those triangles were used in signal decomposition whose edge length was in a given range. On this basis, the mask around the detected component was created. The advantage of the method was the possibility of extracting both vertical and horizontal components, which is superior to VSS. However, the triangulation-based approach works moderately with amplitude modulation in low noise levels. In practice, the edge length of the triangle, including a valid signal, can vary; hence, its discrimination based on a strict length range is insufficient [21,23].

This paper uses the 2D CFAR algorithm to adaptively detect TF distribution local maxima. The proposed approach is similar to that presented in [24], where the CFAR algorithm was also used. However, the components were extracted after parametric morphological operations, which may deteriorate the quality of the reconstruction due to noise extraction or the removal of a fragment of the useful signal. The optimal morphological operation type and the structuring element size may have a crucial influence on the reconstructed signal quality and depend on the propagation environment and the signal itself. Therefore, the processing may need to tune the algorithm to a specific scenario to detect the signal under interest. In the proposed approach, detected regions are grouped using the density-based spatial clustering of applications with noise (DBSCAN), allowing the TF mask to be defined. In order to extract the entire signal content, the mask is modified regarding surrounding spectrogram zeros, which ensures precise signal reconstruction for any waveform character and local disturbances.

The paper has the following structure: Section 2 presents the proposed algorithm. In Section 3, the method is validated using numerical experiments and compared to two techniques known from the literature: VSS [21] and the triangulation-based method [22]. Section 4 presents the application of the real-life radar pulse filtering, and Section 5 concludes the presented findings.

## 2. Algorithm Description

For the complex and continuous signal x(t) and the even and real Gaussian window $h\left(t\right)=\frac{1}{\sqrt{2\pi }\sigma }e^{-\frac{t^{2}}{2\sigma^{2}}}$ with a standard deviation σ, the STFT is defined as follows:

(1) $$F_{x}^{h}\left(t,\omega \right)=\int_{R}x\left(\tau \right)h\left(\tau -t\right)e^{-j\omega \tau }d\tau ,$$

where $j=\sqrt{-1}$. The energy distribution, referred to as a spectrogram, is given as

(2) $$S_{x}^{h}\left(t,\omega \right)={\left|F_{x}^{h}\left(t,\omega \right)\right|}^{2}.$$

In the discrete-time domain, the complex signal is defined as $x\left[n\right]=x\left(\frac{n}{N}\right)$ with n=0,…,N−1, and the Gaussian window h[n] is truncated to be supported on $\left[-M,\ldots ,M\right]$ and obeys 2M+1=K, where *K* is the number of frequency bins. This work assumes that the window length equals the Fourier transform size. For the Gaussian window, which theoretically has an infinite length, the assumption amounts to elongation of the window with samples very close to 0, which can be regarded as zero-padding. The discrete STFT variant of (1) is given as [25]

(3) $$F_{x}^{h}\left[m,k\right]=\sum_{n\in Z}x\left[n\right]h\left[n-m\right]e^{-j2\pi \frac{k(n-m)}{N}},$$

where k=0,…,K−1, and m=0,…,N−1. The analogous distribution for (2) follows as

(4) $$S_{x}^{h}\left[m,k\right]={\left|F_{x}^{h}\left[m,k\right]\right|}^{2}.$$

**Figure 1:**
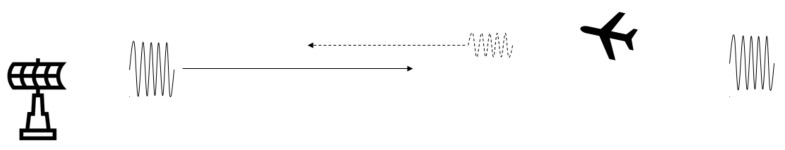
A simplified idea of the radar system. Solid arrow—transmitted signal, dotted arrow—reflected signal.

In this paper, the TF distribution obtained using (1) is processed in the same way as in radar. In short, a radar system is a device that sends an electromagnetic signal into space and then receives signal reflections from obstacles. The transmitted and received signals are compared, which allows the radar to estimate a target’s range (comprising the delay between the transmitted and received signal) and its radial velocity (by analyzing the Doppler shift presentin the received signal). A simplified diagram of the radar operation is shown in Figure 1.

In practice, the receiver gathers multiple reflections not only from the target, but also from stationary objects such as buildings, trees, etc. Thus, spikes resulting from signal reflection from moving targets in the presence of noise are extracted using the CFAR algorithm, allowing an adaptive threshold to be obtained [26,27], and then, a binary decision is made [26]. The main advantage of the CFAR algorithm is the ability to adapt the threshold level to different kinds of noise, such as Gaussian and alpha stable, among others [28]. For the distribution X(t), one may write the detection condition as

(5) $$D\left(|X(t)|\right)=\left\{\begin{array}{ll} 1 & \mathrm{if}\;|X(t)|\geq T,\\ 0 & \mathrm{otherwise}, \end{array}\right.$$

where *T* is the regularized threshold governed by

**Figure 2:**
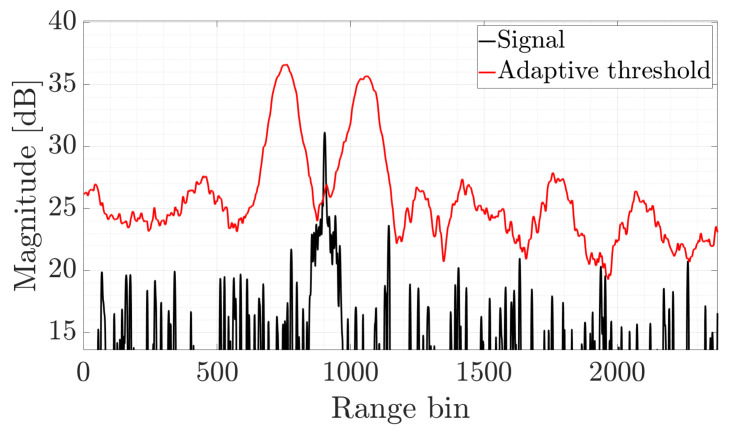
An example of a CFAR detection result using a real-life radar signal after range compression.

(6) T=RC,

where *C* is the noise estimate depending on the CFAR algorithm variant (in the literature, one can find a wide range of descriptions of different CFAR techniques [26,27], and for the sake of clarity and consistency, they are not duplicated in this work). *R* is the factor manipulated by the probability of false alarm $P_{f}$, so that

(7) $$R=N_{T}\left(P_{f}^{-\frac{1}{N_{T}}}-1\right),$$

where $N_{T}$ is the number of points used to estimate the clutter level. An example of adaptive target detection in a real-life radar is shown in Figure 2. The way in which the CFAR algorithm was applied is typical in radar; namely, after the so-called range compression, the target echo can be extracted from the noise. The result shows a one-dimensional CFAR detection performance to demonstrate its main properties. The principle is the same for a bivariate case; however, it would be much more difficult to illustrate. As can be observed, the threshold is adaptively adjusted based on the local noise estimate. The threshold rapidly grows and then drops for the apparent target around the 900 range bin, which yields the target detection. The same procedure is applied to the spectrogram in the proposed algorithm.

**Figure 3:**
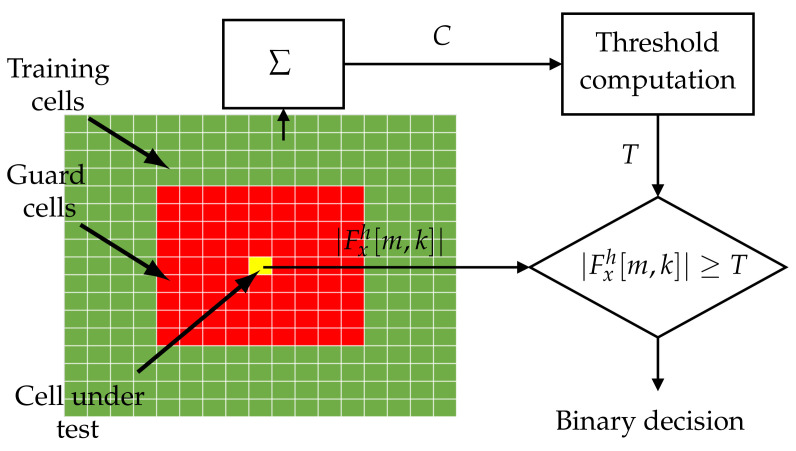
The principle of the CFAR algorithm’s operation.

Since the spectrogram is a two-dimensional distribution, the CFAR algorithm is two-dimensional as well. In this work, vertical and horizontal training cells are denoted as $N_{T}^{V}$ and $N_{T}^{H}$, respectively, $N_{T}=N_{T}^{V}+N_{T}^{H}$, and vertical and horizontal guard cells are given by $N_{G}^{V}$ and $N_{G}^{H}$, respectively. The idea behind the CFAR algorithm is illustrated in Figure 3.

In this paper, for the STFT analysis, two sets are created around each point of the $|F_{x}^{h}\left[m,k\right]|$ distribution (yellow cell under test). The first one is composed of so-called guard cells marked in the diagram in red. They are not considered during processing since they may influence noise estimates when the strong component occurs. The second group (in green) is built of the training cells. They are used for noise level estimation, usually by averaging or statistical operations. The process is carried out for each [m,k] of the STFT, and as a result, a binary detection map for the whole distribution is obtained according to (5). In this work, the greatest of cell averaging (GOCA) CFAR is used due to its better properties with heterogeneous distributions [26]. However, any other detector can be applied.

Detected points are clustered using the DBSCAN method. In short, this is an efficient, non-parametric technique of grouping points that are closely located and labeling as outliers those groups that lie outside [29]. The algorithm divides points (in this work, points resulting from the CFAR detection) into three sets: core point (which the clustering process starts with); border point (when no more points can be grouped into it); noise point (does not belong to any cluster and two other groups). STFT regions where any coherent structure occurs provide closely packaged points after CFAR detection that can be easily clustered using the DBSCAN algorithm. Detected regions (sub-domains) are sorted concerning the signal energy on the spectrogram. In the CFAR algorithm, $P_{f}$ selection comprises a trade-off between weak signal extraction and incorrect detection and is usually in the range of $P_{f}\in \left(10^{-6},10^{-3}\right)$. Thanks to signal clustering using the DBSAN method, false detections are not destructive for component retrieval. Since component energy is taken into account when sorting, all modes stronger than the noise appear higher than the clutter in the sorted list of sub-domains. To ensure a component’s extraction, $P_{f}$ can be greater than in radar, which is a meaningful advantage and results in robustness when joined with the DBSCAN method. The selection of appropriate processing parameters (e.g., the number of guard and training cells, the probability of false alarm) depends on the application, the number of time and frequency bins of the STFT, and the noise level.

The selected sub-domains allow TF masks to be created. As mentioned in this section, the CFAR algorithm assumes a given regularized threshold to detect the spikes. On the other hand, a part of the signal below the threshold is pruned off. Thus, some part of the signal information is lost, since, in practice, the threshold is non-zero. The problem does not matter in radar since the precise signal reconstruction is not needed in most situations, and only the target range and/or velocity are estimated. In signal extraction from the TF plane, the cut-off part of the distribution results in imprecise reconstruction due to the energy pruning. However, as shown in [22,30], the STFT is fully characterized by spectrogram zeros. Following the idea Flandrin and Bardent et al., the STFT given by (1) can be expressed as

(8) $$F_{x}^{h}\left(t,\omega \right)=e^{-\frac{{\left|z\right|}^{2}}{4}}F_{x}\left(z\right),$$

where

(9) $$F_{x}\left(z\right)=\int_{R}A\left(z,u\right)x\left(u\right)du,$$

is the Bargmann transform [31], whose kernel takes on the following form:

(10) $$A\left(z,u\right)=\pi^{-\frac{1}{4}}e^{-\frac{u}{2}-juz+\frac{z^{2}}{4}}.$$

Equation (9) is a function of positive order and admits the Weierstrass-Hadamard factorization:

(11) $$F_{x}(z)\propto \prod_{n=1}^{\infty }\left(1-\frac{z}{z_{n}}\right)e^{\frac{z}{z_{n}}+\frac{1}{2}{\left(\frac{z}{z_{n}}\right)}^{2}},$$

where $z_{n}=\omega_{n}+jt_{n}$ are zeros of the Bargmann transform (9). These properties were used for component retrieval through the connection of spectrogram zeros using the Delaunay triangulation. However, sensitivity to noise, clutter, and amplitude disturbances make the approach difficult to apply in, e.g., radar systems [21,23]. However, a zero distribution is used in this work to spread the TF mask and extract the entire signal from the STFT. Doing so for the detected signal component clustered using the DBSCAN method, one has to find surrounding spectrogram zeros and extend the mask in order to precisely distinguish the specific signal mode. After that, the STFT can be multiplied by the mask, and the inverse STFT can be applied, obtaining a precise signal reconstruction.

The proposed algorithm can be divided into the following steps:

1. Compute the STFT $F_{x}^{h}\left[m,k\right]$.
2. Detect protruding regions (sub-domains) from $|F_{x}^{h}\left[m,k\right]|$ using the adaptive CFAR thresholding.
3. Group the detected points into clusters using the DBSCAN algorithm.
4. Sort clusters comprising their energy.
5. For each desired component, spread the mask to the nearest zeros of the spectrogram $S_{x}^{h}\left[m,k\right]$.
6. Apply the STFT masking.
7. Retrieve the signal back in the time domain using the inverse STFT.

**Figure 4:**
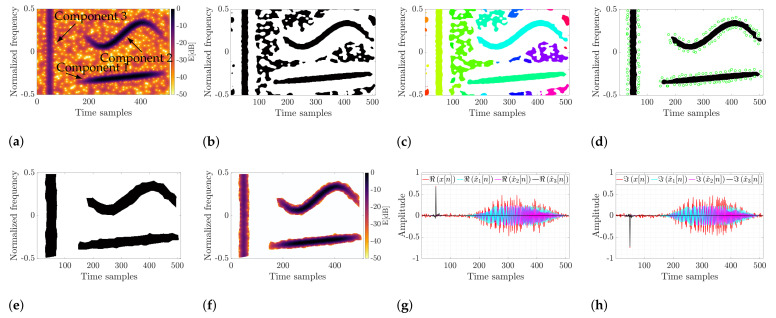
Simulation results for the multicomponent amplitude- and frequency-modulated signal. (**a**) Simulated signal; (**b**) Detected components; (**c**) Clustered components; (**d**) Initial masks with zeros; (**e**) Spectrogram after masking; (**f**) Final TF masks; (**g**) Extracted modes (real part); (**h**) Extracted modes (imaginary part).

The outcomes for the exemplified multicomponent signal with vertical, horizontal, and amplitude-modulated terms are presented in Figure 4. In Figure 4a, the spectrogram is illustrated. Three entirely different modes contaminated by noise are apparent. After the adaptive thresholding, detected points are presented as black regions in Figure 4b. Next, they are clustered as shown in Figure 4c, where a different color specifies each sub-domain. Next, the masks are spread to their adjacent spectrogram zeros for a defined number of components. The masks (in black) with detected zeros (green points) are shown in Figure 4d, and their final form is depicted in Figure 4e. The spectrogram after applying the mask looks like the one shown in Figure 4f, and the real and imaginary part of the retrieved components is shown in Figure 4g and Figure 4h, respectively. As shown, the method allows for precise signal retrieval from the TF plane regardless of its character. A typical problem known in the literature is extracting various types of signals, e.g., horizontal, vertical, and mixed terms. In the proposed approach, the signal character does not matter, and the extracted signal can be of any nature, e.g., impulsive spike, chirp, harmonic term, or any non-defined TF structure such as a telecommunication signal. The main limitation is the difficulty of separating the intersecting components, and such signals are not analyzed in this work. The issue requires further in-depth analysis. In the next section, the method is compared to the techniques known from the literature.

## 3. Simulations

The proposed method was compared to two other well-defined techniques in the literature. The first is the one proposed by Flandrin [22] with a default range of the triangle edge length for noise $e_{l}\in \left[0.2,2.2\right]$. The second one was VSS, whose definition is as follows [20]:

(12) $$S_{x}^{h}\left(t,\omega \right)=\int_{R}F_{x}^{h}\left(t,\Omega \right)\delta \left(\omega -{\hat{\omega }}_{x}\left(t,\Omega \right)\right)d\Omega ,$$

where ${\hat{\omega }}_{x}\left(t,\omega \right)$ is the frequency reassignment operator. In this work, one of the most precise and efficient approaches to VSS was used, namely the technique called the enhanced first-order VSS (EVSS1) proposed in [21]. For mode extraction from the synchrosqueezed STFT, the technique initially proposed in [32] and implemented in [33] was applied. The approach is based on the local minimum computation of the formula:

(13) $$E_{x}\left(\psi_{1},\ldots ,\psi_{K}\right)=\sum_{k=1}^{K}-\int_{R}|{\mathrm{TF}}_{x}{\left(t,\psi_{k}\left(t\right)\right|}^{2}+\lambda \psi_{k}^{\prime }{\left(t\right)}^{2}+\varepsilon \psi_{k}^{\prime \prime }{\left(t\right)}^{2}dt,$$

where *K* stands for the known number of components $\psi_{k}\left(t\right)$ extracted from the distribution ${\mathrm{TF}}_{x}$. According to [34], λ and ε were set to 0 in the analysis due to their irrelevant influence on ridge detection. Additionally, in all analyzed cases (simulated and real-life) and for all methods in question, the width of the analysis window was selected to minimize the Rényi entropy [35] or, for signals with several completely different modes, to ensure a relatively constant resolution for all components. Furthermore, in all experiments, the sliding step of the window was assumed to be unitary since it streamlines the inverse STFT computation; however, this value is not fixed and can be easily manipulated.

**Figure 5:**
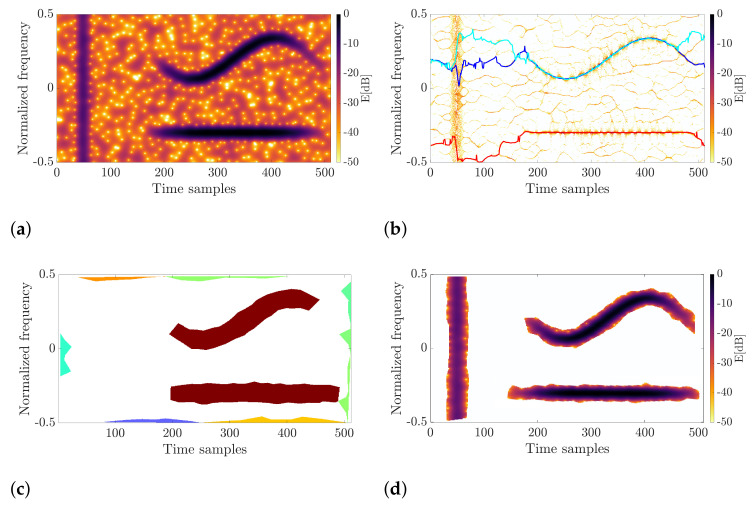
Comparison of different methods for signal retrieval from the time–frequency distribution. For vertical synchrosqueezing and the triangulation-based method, lines and regions with different colors express detected components. (**a**) Spectrogram; (**b**) Vertical synchrosqueezing; (**c**) Triangulation; (**d**) Proposed method.

A comparison of the results for the known techniques and the proposed algorithm is shown in Figure 5. As can be seen, none of the techniques from the literature allow for extracting all components sufficiently from the spectrogram depicted in Figure 5a. For example, vertical synchrosqueezing (Figure 5b) concentrated the harmonic term and sinusoidal chirp well. However, the pulse at the beginning was smeared, and its reconstruction is impossible. Known algorithms are unable to distinguish incorrectly concentrated components. Thus, the common techniques are of limited usability. The vertical component was not even detected for the approach based on spectrogram zero triangulation, as shown in Figure 5c, where different colors represent detected signal modes. As presented in Figure 5d, the proposed method can precisely retrieve all of these components, which is the superiority among the methods known in the literature. As already mentioned, the proposed technique does not assume a signal model; thus, any structure can be extracted regardless of its nature.

To assess the proposed method’s usability quantitatively, let us consider a nonlinear chirp with sharp nonlinearity at its ends. Such a kind of signal is commonly used in military and civilian radar systems, e.g., air traffic control (ATC) [21]. The simulated signal x[n] of a length *N* and sampled with a rate $f_{s}=12.5$ MSa/s is defined as

(14) $$x\left[n\right]=A_{x}\exp\left(j2\pi \left[\alpha \frac{{\left(n-\frac{N}{2}\right)}^{2}}{2}+\gamma \frac{{\left(n-\frac{N}{2}\right)}^{10}}{10}\right]\right)+w\left[n\right],$$

where n∈[0,…,N−1], $\alpha =1.5\cdot 10^{11}\frac{\mathrm{Hz}}{s}$ and $\gamma =2\cdot 10^{50}\frac{\mathrm{Hz}}{s^{9}}$ are the chirp rate and the nonlinear frequency modulation term, respectively, $A_{x}$ is the unitary amplitude, and w[n] is the additive white Gaussian noise. The pulse was multiplied by the Tukey window to simulate a non-zero duration of the rising and falling edges at the beginning and the end of the waveform (amplitude modulation), and after this operation, the signal duration was *T* = 30 μs.

In order to define the reconstructed signal quality, the reconstruction quality factor (RQF) was used for all considered methods [23]:

(15) $$\mathrm{RQF}=10\log_{10}\frac{\sum_{n=0}^{N-1}{\left|x\left[n\right]\right|}^{2}}{\sum_{n=0}^{N-1}{\left|x\left[n\right]-\hat{x}\left[n\right]\right|}^{2}},$$

where x[n] is the known and pure waveform and $\hat{x}\left[n\right]$ is its retrieved form. The calculations were carried out for six signal-to-noise ratio (SNR) values SNR={5,10,15,20,25,30} dB and averaged over 100 Monte Carlo simulations for each method. The STFT was computed using the fast Fourier transform algorithm with 512 points and the Gaussian window with a normalized standard deviation σ=8. In this case, $P_{f}=0.4$ results in noise detection, but allows for the extraction of amplitude-varying and weak components’ extraction. The remaining parameters are as follows: training cells $N_{T}^{V}=16$, $N_{T}^{H}=16$, guard cells $N_{G}^{V}=16$, $N_{G}^{H}=16$.

**Figure 6:**
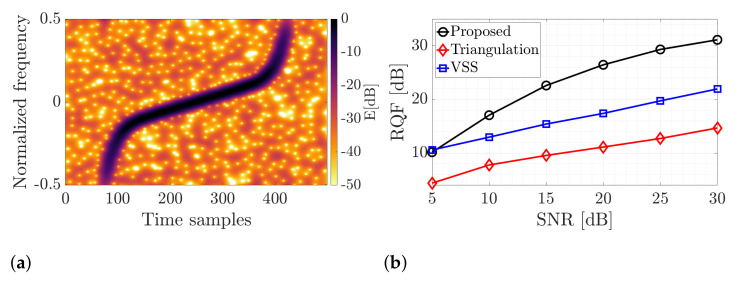
Simulation results for the nonlinear chirp signal. (**a**) Spectrogram; (**b**) RQF.

Figure 6 presents an example of a spectrogram and the results of signal retrieval. As shown, the proposed algorithm allows for the best signal reconstruction over the validated techniques. One can even extract vertical components thanks to operating on a non-concentrated distribution. Furthermore, the independence of the length between spectrogram zeros makes the introduced technique efficient and robust in noise and amplitude modulation. The RQF is higher even by 15 dB compared to the triangulation-based approach and 10 dB compared to VSS (differences for SNR=30 dB). The signal reconstruction results were improved mainly thanks to the non-parametric analysis of the signal distribution on the TF plane. Therefore, the signal model is not used in the extraction process, as is usually the case with methods from the literature. In the proposed solution, the signal may contain any component, and the proposed technique does not assume a specific function defining the phase.

## 4. Real-Life Signal Analysis

Signal decomposition is an issue in various applications, and the proposed algorithm can be used widely. As a representative example, frequency-modulated radar chirps were considered. The main goal was to extract only the direct pulse, whilst maintaining amplitude, phase, and frequency dependencies unaltered. In this case, a simple inverse STFT is useless. As a result, one would obtain the unmodified signal (the signal was recorded in the time-domain so that the inverse STFTwill result in the same signal). Thus, the proposed algorithm can be practically used in such an application.

### 4.1. Nonlinear Frequency Modulated Pulse

**Figure 7:**
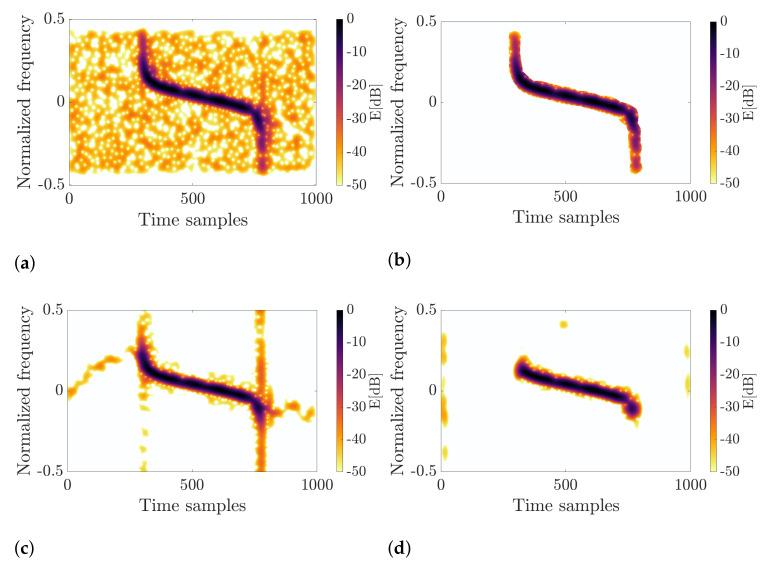
Input real-life radar pulse and the retrieval results for three methods under consideration. (**a**) Input signal; (**b**) Proposed method; (**c**) Vertical synchrosqueezing; (**d**) Triangulation method.

The first signal originates from the ATC radar system located at Warsaw’s Chopin Airport, Poland. The sampling rate during the data collection was $f_{s}=40$ MHz; then, the signal was downsampled so that the final $f_{s}=5$ MHz. The selected pulse (shown in Figure 7a) was processed in the same way as during the simulations. The window standard deviation, in this case, was σ=10, and the processing was carried out for 512 points of the FFT. In this case, the CFAR parameters were as follows: $P_{f}=0.4$, $N_{T}^{V}=12$, $N_{T}^{H}=8$, $N_{G}^{V}=24$, and $N_{G}^{H}=16$. Since the transmitting radar was non-cooperative and the waveform signature is unknown, the RQF cannot be computed in this case. The signal under consideration and the processing outcomes are presented in Figure 7.

The results clearly show differences between the retrieved pulses. After processing using the method proposed in this work, the entire waveform was extracted, including its linear and nonlinear terms, as shown in Figure 7b. With the VSS-based approach, vertical components (transitions and highly modulated terms) are lost because they cannot be concentrated, and therefore, energy is spread across the plane instead of being focused on the instantaneous frequency ridge. Therefore, the major part of the reconstructed pulse consists of the linear chirp, and the whole signal information was not captured. A similar outcome was achieved for the spectrogram zero triangulation method. In this case, pruning of the pulse ends results from the assumption on the triangle edge length. As can be observed, the edge length for noise $e_{l}\in \left[0.2,2.2\right]$ does not allow for dealing with all components, especially those with rapid frequency and amplitude modulation.

**Figure 8:**
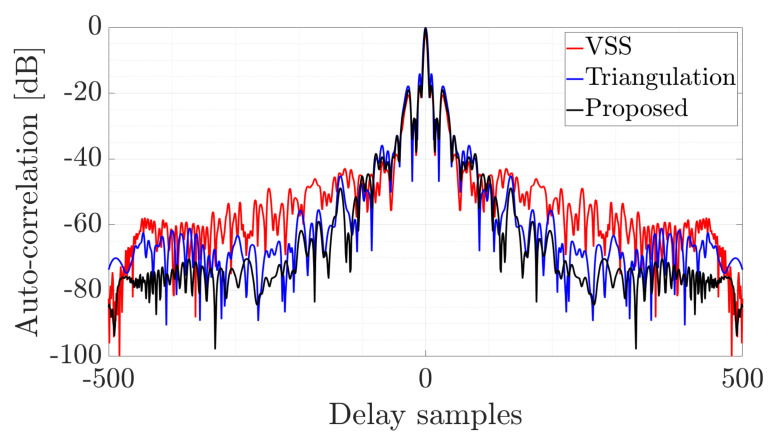
Auto-correlation of the reconstructed NLFM pulses.

The reconstructed pulse can be used in a passive radar that uses another active radar as a source of illumination [36] or for specific emitter identification in electronic warfare. The quality and correlation properties of the pulse have a key influence on the radar’s detection capability. The better the reconstruction quality, the greater the radar’s ability to detect the target. The results of the auto-correlation of the reconstructed pulses are shown in Figure 8.

As can be seen, the best capabilities were obtained for the proposed method since the side-lobes have the lowest value, while the peak is high and narrow. The main factor influencing the quality of the recovered pulse is the possibility of extracting vertical components at the ends of the transmitted signal. Only the proposed method allows complete pulse extraction, including the part with linear and nonlinear frequency modulation. Furthermore, resistance to local amplitude variation is achievable with the proposed approach. It should be noted that local amplitude deviations resulting from various factors such as the nonlinearity of the radio frequency chain, noise, and multipath propagation influence the amplitude distribution on the spectrogram. That has a crucial impact on the efficiency of methods known from the literature [21,22], which are characterized by inferior properties to those of the presented algorithm. This shows the effectiveness of the proposed method and the possibility of its use in practical systems.

### 4.2. Linear Frequency-Modulated Pulse with Strong Multipath Interference

**Figure 9:**
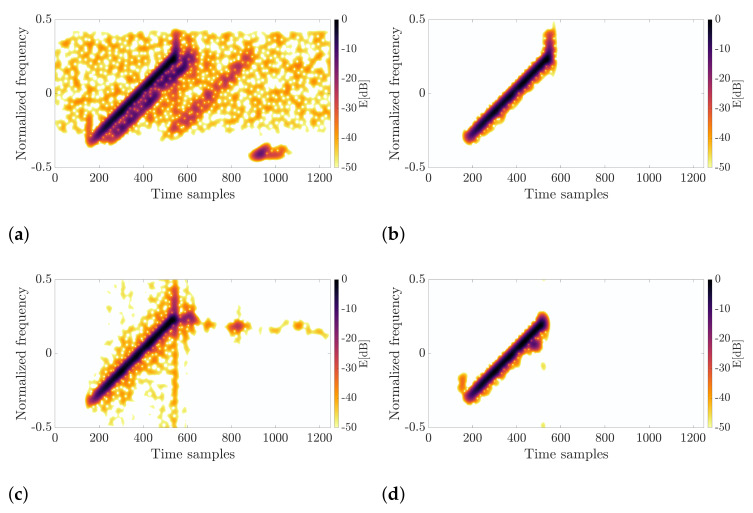
Results for the second signal. (**a**) Input signal; (**b**) Proposed method; (**c**) Vertical synchrosqueezing; (**d**) Triangulation method.

**Figure 10:**
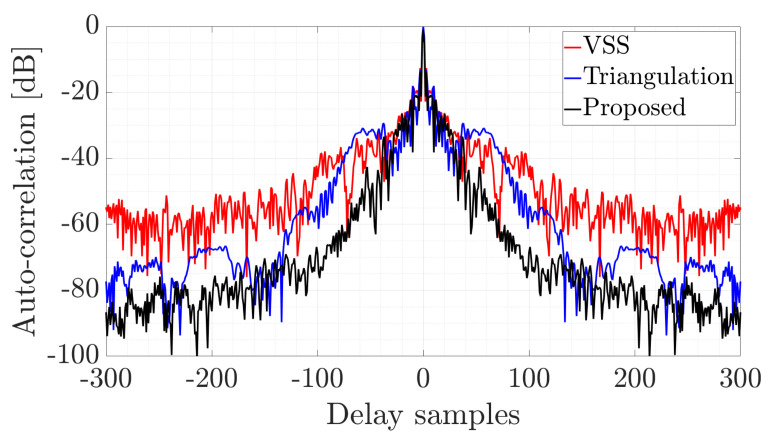
Auto-correlation of the reconstructed LFM pulses.

The second signal was transmitted by the medium-range radar and recorded with a similar setup as in the NLFM case. The results are illustrated in Figure 9. Apart from the strong direct signal in Figure 9a, delayed copies of the pulse are also apparent in the receiver. For the proposed method, the entire pulse was correctly retrieved with preserved amplitude, phase, and frequency. For the VSS-based approach, the pulse was recovered, but the surrounding noise disturbed its quality. In addition, the component extraction method contributed to the extraction of a certain amount of noise across the entire time axis. The result is shown in Figure 9c. In the method using the triangulation of spectrogram zeros, the results of which are presented in Figure 9d, the reconstruction is also worse than for the proposed method. The impulse was shortened, and some multipath disturbances were also extracted. The best result was obtained for the new method presented in this article, as shown in Figure 9b, where the precisely retrieved signal is shown. It is worth noting that the precise pulse extraction was achievable thanks to the use of the CFAR algorithm, which estimates the noise level and defines the threshold adaptively. The nature of the disturbance may have a different distribution than Gaussian for the multipath effect just after the direct pulse. As can be seen, the CFAR algorithm correctly assessed its level giving a correct threshold value. For the proposed algorithm, the normalized standard deviation of the window was σ=10, and the processing was performed for 512 points of the FFT and the following CFAR parameters $P_{f}=0.4$, $N_{T}^{V}=8$, $N_{T}^{H}=8$, $N_{G}^{V}=8$, and $N_{G}^{H}=8$. As in the previous case, the pure waveform character was unknown to the author; thus, the RQF cannot be calculated. Therefore, the auto-correlation function was computed and is shown in Figure 10. It is clear that the pulse retrieved for the proposed technique is characterized by the best performance from the radar point of view. Apart from the lowest side-lobe level, the peak is the narrowest, allowing target detection.

## 5. Conclusions

This paper presented a novel signal decomposition approach to component retrieval from the STFT with examples supporting its effectiveness. The superiority of the presented finding relies on the unsupervised ability to extract differently oriented waveforms, e.g., bursts, transient, nonlinear chirps, harmonic terms, and signals with amplitude modulation. The proposed approach’s properties were confirmed by thorough numerical experiments using simulated signals and real-life radar pulses. Future research should cover the proposed algorithm’s extension to the analysis of multicomponent signals with crossing modes and rapidly oscillating instantaneous frequency. In another direction, work should involve CFAR algorithm parametrization comprising signal and processing parameters.

## Data Availability

Not applicable.
